# 34. Biopsy proven primary angiitis of the central nervous system in a patient with rheumatoid arthritis

**DOI:** 10.1093/rap/rky033.025

**Published:** 2018-09-20

**Authors:** Sabreen Ali, Warren Shattles

**Affiliations:** Rheumatology, East Surrey Hospital, Redhill, UNITED KINGDOM

**Introduction:** Central nervous system manifestations in rheumatoid arthritis (RA) are rare and serious, and present a complex diagnostic and management challenge.

**Case description:** A 52 year old lady presented acutely to the emergency department after being found on the floor by a neighbour. She was thought to have had a long lie of three days, and was unable to fully recall the events that led to her fall. She had a background of seropositive rheumatoid arthritis and was otherwise well. Her rheumatoid arthritis was well controlled on methotrexate, leflunomide and prednisolone, and she had previously used anti-TNF (tumour necrosis factor) biologic therapy in the form of certolizumab, which had been stopped due to inefficacy. Her inflammatory markers had been quiescent for a long time prior to her presentation, most recently up until 12 days prior to admission. On examination she had a blood pressure of 153/84 mmHg; other observations were normal. She had a left facial droop and her speech was slightly slurred, with a mild decrease in power of her left upper limb. Lower limb examination was limited because of pain in both hips. Sensory examination was intact. Cardiorespiratory examination was normal, as was her abdominal examination. She was thought to be mildly confused. Her blood tests revealed a leucocytosis of 17.3 x 10^9^/L with a neutrophil count of 14.3 x 10^9^/L. Her C-reactive protein was elevated at 188 mg/L. She also had a raised creatine kinase (CK) of 10, 523 u/L, and an acute kidney injury with a urea of 15.9 mmol/L and creatinine 117 mmol/L. Her chest x-ray did not show consolidation, and pelvic x-ray ruled out fracture. Urine dip was positive for blood and protein, but negative for nitrites and leucocytes. Computed tomography (CT) of her brain reported a small acute right frontal parenchymal bleed with surrounding oedema and local mass effect. There was no associated midline shift. A neurosurgical opinion was sought, and conservative management was advised at this stage. She was therefore admitted to the stroke ward for further care. On focussed questioning it was noted that the patient had been feeling unwell for the preceding three weeks with nausea, neck pain and morning headache. She described no fever, photophobia or rash. Her CK, urea and creatinine levels responded to IV fluid rehydration, and she remained clinically stable. Inflammatory markers also improved without antibiotics. Pre and post contrast magnetic resonance imaging (MRI) scans of her brain were obtained later that day, which confirmed a right frontal haematoma with no pathologically enhancing mass underlying it. The vasogenic oedema surrounding the haematoma was noted to be quite extensive. An area of evolving ischaemia was noted in the thalamus, as well as periventricular and corpus callosum high signal change thought to be out of keeping with simple small vessel disease given the extent of the distribution, as well as the age of the patient. A CT angiogram of the aortic arch and carotid arteries was found to be unremarkable. A lumbar puncture was performed, which showed a normal opening pressure of 14.5 cm of water, with 59 white cells per uL and 94 red blood cells per uL. Cerebrospinal fluid (CSF) protein was elevated at 0.73g/L. There was a 100% lymphocytosis on fluid analysis, and viral polymerase chain reaction (PCR) and bacterial culture were both negative. A neuroradiology opinion suggested a differential diagnosis of cerebral vasculitis secondary to RA, or potential underlying malignancy. On rheumatology review, musculoskeletal examination revealed chronic changes of RA in the metacarpophalangeal joints bilaterally, without active synovitis. There was mild effusion in the right knee without erythema or joint line tenderness. There was no evidence of digital, nail or skin vasculitis and no pleural or pericardial rub was auscultated. In summary, there was no evidence of systemic RA activity. Given the lack of other systemic features such as a mononeuritis multiplex or vasculitic skin lesions, an isolated cerebral vasculitis associated with RA was considered an unlikely diagnosis, and a malignant lesion or primary angiitis of the central nervous system were considered as possible differential diagnoses. A repeat MRI did not show any change to the diffuse infiltrative process in the right frontal lobe, white matter, right basal ganglia, anterior aspect of corpus callosum and white matter of the left frontal lobe. There was a mild decrease in the size of the large haemorrhage in the right frontal lobe, with newly developed small punctate bleeds in the parafalcine region of the same lobe, favouring an active process. In order to clarify the diagnosis, the patient was transferred to a tertiary centre for brain biopsy. The neuropathologist concluded that biopsy appearances were consistent with that of a granulomatous vasculitis suggestive of primary angiitis of the central nervous system (PACNS). Small vessels including arteries in the leptomeninges, cortex and white matter showed destruction, occlusion and infiltration of their walls by plump histiocytes, lymphocytes and occasional multinucleated cells. Immunostaining results were also congruent. Other potential causes of granulomatous inflammation including neurosarcoidosis, tuberculosis and fungal infection were in the differential but thought to be less likely.

**Discussion:** Vasculitis can involve both the central and peripheral nervous systems, with central nervous system (CNS) vasculitis being far rarer. CNS vasculitis is broadly categorised into two entities, PACNS and secondary CNS vasculitis associated with a systemic disorder. The latter group includes CNS vasculitis secondary to systemic autoimmune diseases (systemic lupus erythematosus, rheumatoid arthritis), idiopathic vasculitides (polyarteritis nodosa) and non-autoimmune disorders (infection, drugs and malignancy). Cerebral rheumatoid vasculitis is exceedingly uncommon in RA, and leads to significant mortality especially in the presence of a concurrent systemic vasculitis. Central nervous system involvement in RA has been previously reported mostly in seropositive patients with longstanding active and erosive disease, especially with subcutaneous nodules and extra-articular features. Our patient did not have nodules or extra-articular features of RA and had well-controlled disease. This in combination with the extent of oedematous change on her imaging made rheumatoid vasculitis a less likely diagnosis, although it could not be ruled out without a brain biopsy. Primary angiitis of the central nervous system was therefore considered in light of these factors. Brain biopsy remains the gold standard for diagnosis of PACNS, and usually demonstrates a lymphocytic inflammatory reaction with variable numbers of plasma cells, histiocytes, neutrophils and eosinophils (4). Focal and segmental granulomatous lesions are found in the majority of cases. Necrotising features or a leucocytoclastic vasculitis are more in favour of a secondary vasculitis related to rheumatoid arthritis.

**Key Learning Points:** CNS vasculitis can be primary or secondary, and careful evaluation and work up is required to make the diagnosis. The presence of a potentially confounding underlying condition should not rule out the diagnosis of PACNS, and careful assessment of the patient is required to exclude features of systemic vasculitis. Brain biopsy is the gold standard for the diagnosis of cerebral vasculitis, and adverse event risk is generally low in experienced centres.

**Disclosure: S. Ali:** None. **W. Shattles:** None.

